# The Meeting in Wigmore Hall, London

**Published:** 1919-03-01

**Authors:** 


### THE MEDICAL PROFESSION ON A TRADE UNION BASIS.

### The Meeting in Wigmore Hall, London.

In response to a circular issued by the Medico-Political Union to the members of the medical profession in London and near vicinity, headed "The Crisis in the Medical Profession," a very large gathering mustered in Wigmore Hall on Sunday afternoon last, 23rd ult., the hall being full, including a number in the gallery. The chair was occupied by the President of the Union, Mr. Frank Coke, F.R.C.S.

The Chairman said every section of the profession had been invited, because, with the imminent changes foreshadowed by the Ministry of Health Bill now on the table of the House of Commons, it was imperative that every branch of the profession should be able to gain a hearing for itself and be able to make itself felt by force of argument. He wished to dispel one or two glaring fallacies with regard to the Medico-Political Union. They were not Bolshevists, nor Socialists, nor any crank formation, but general practitioners carrying on a hard existence. They had a strong objection to using force, but that might be the only means of regaining their professional dignity and independence. No strike could be called by the Union without a ballot being taken of the views of every member. There was never any idea of striking against the sick person himself. Still, the worm might eventually have to turn. Every branch of the profession was welcomed into the organisation, and they would be pleased to confer with other bodies for mutual benefit, but they did not intend to hand over one iota of the responsibility with which they in the Union had been entrusted: their desire was to see the work through themselves. The majority of the members were on Panel work, and most of the legislation of the past had affected that unfortunate class, as would that of the future. Each branch of the profession should be able to get a hearing for its own particular needs.

Dr. J. Alexander Angus proposed the following resolution:—

"That, in view of the far-reaching changes inevitable in the Medical Services of this country consequent on the coming Ministry of Health, it is essential that the profession should be solidly and democratically organised on a trade union basis, to enable it to negotiate effectively with the Government in the interests of the community no less than in those of the profession."

He declared that the profession had met on the threshold of events of the greatest import to itself. On the one hand, this might mean an enhanced and improved profession; or, on the other, disaster and ruin to the general practitioner and those who, like consultants, depended on the general practitioner. In their attempts to improve the profession they had met with no response from the powers that be, and with no evidence of real sympathy. They had to deal with a political authority, and the politician valued his adversary just in proportion to the power of the latter to do him an injury or ruin his pet schemes. This was the crucial time for the profession to protect its interests. If it were not done, there might be but little for it to protect in the future. They cordially welcomed the advent of the Ministry of Health, as well as Dr. Addison in the highest capacity in association with it. If justly and honestly administered it might be productive of the greatest good. But if administered in the same way as the Insurance Act was it would be of no good to the profession, and the far-reaching powers of the Ministry might be exercised to the profession's detriment. It was not lowering to the dignity of a profession with such a great power for good to the community that it should use trade-union laws. The efficacy of the new Ministry would depend on the institution of a Medical Advisory Committee, hence the constitution of that committee must be truly representative. It must be a statutory committee. The Advisory Committees of the National Health Insurance were not statutory, and they were a mockery and a sham. It was not contended that the Minister of Health must necessarily act on the advice of the Advisory Committee: if he was responsible to the Government and the country he must have liberty; but, whether he took the advice of that Committee or not, he must consult it. The opinion of the Committee must be on record, so that if the Minister expressed a policy in opposition to it the responsibility would be his, and he must be prepared to defend himself. And there must be a contract or agreement between the profession and the Government, and, for God's sake, let it be a real contract this time! The profession had been tricked before. What was called the agreement between the Commissioners and the medical practitioners working the National Insurance Act was no agreement; it was peculiarly prepared that one party to it—the Commissioners—could vary the agreement at will, but the profession could do nothing of the kind. There must be no alteration of terms, except by the absolute agreement of both parties to the original contract. He proceeded to refer to the extraordinary action of the Local Government Board with regard to notification of infectious disease. Powerful as Mr. Lloyd George might be, and great as his majority in the present House of Commons might be, he dare not have treated a deputation of working men as medical men were treated in connection with the Infectious Diseases Act. (Applause.) With regard to the suggestion for a whole-time State Medical Service, he believed the profession was unanimous in regard to that. It would mean good-bye to the family physician, to the doctor-friend; it meant the breaking up of all the traditions of the profession, and the stereotyping which would be hateful to the profession generally. Consultants must realise that their interests were bound up with those of the practitioner, and both must stand or fall together. In the ultimate extremity there was only one thing to do—namely, to use the trade union law of combination for the purpose of extracting from a political authority a just measure of that which was their right.

Dr. E. H. Stancomb (Southampton) seconded the resolution. He said the Association was registered under the Trades Disputes Act, 1915, as "The Panel Medico-Political Union." He hoped it would include the great mass of the profession, who would very soon be awakened to the fact that they needed a real live legalised constitutional method of defence, for their interests were seriously challenged. It was realised that the new conditions required new methods. The Association aimed at two things: the real and permanent good of the community, and the maintenance of the dignity, status, and proper treatment of the medical profession. He would not say how far other organisations, formed under other conditions, had been adapted to meet the new circumstances now operative, but it was felt that a more active and more stringent defence was now needed. One thing which the miners, by their power exerted through numbers, achieved was the recognition that their claim could not be discourteously and abruptly side-tracked, but must receive fair and courteous consideration, whatever the result might be. The Medico-Political Union stood to bring such pressure on the Government as would ensure equal consideration of the claims of the profession. They did not intend to rush into the dangers and difficulties of a strike; that could only be achieved by the plebiscital decision of the members, and all the members of the Council would regard a strike as a much-to-be-deplored and infinitely-to-be-delayed resort. If they did strike, they would be justified in demanding equal pay for equal work. Why were people willing to pay Charlie Chaplin £300,000 a year to make them laugh, while they paid him, the speaker, only £600 a year to keep them alive? (Laughter.) Was it alleged that one was worth 500 times as much as the other? Lawyers were very numerous in Parliament; they had at their back the laws they had made, and theirs was one of the tightest and most powerful trades unions the world had ever seen. Yet the medical man, trained and educated to build up and reconstruct humanity, was, apparently, to be content with the crumbs which fell from the rich man's table; he was to be the self-sacrificing, good-natured gentleman, and that idea was played up to. Where should we have been in the war but for the intensive use which was made of the Medical Service to prevent the waste of the Forces through unnecessary sickness? He was prepared to give his services under certain conditions which were consistent with the dignity and work of an educated and trained professional man; and he must have an assurance that when his contract had been made he would not be let down upon it. The only way in which to get anything out of the Government was to be equipped with the greatest possible protective force. Trade-unionism was under Government blessing: it had been recognised and legalised. It was absolutely democratic, but there was a tendency on the part of some to say it was not gentlemanly, and that the profession should be above it. His answer was that they would be above it when those who were above doctors would stop sweating and exploiting them. Employers combined as well as workers. In adopting trade-unionism for the time being doctors were not sinking to the level, but were rather rising to the altitude. He believed unity would come to the profession in the very near future, when the colossal importance of its function in the State was recognised, and it would be recognised that there was ample room for the triumvirate—its science, its trade-union section, and its Parliamentary representation. He hoped this meeting would prove a great help towards securing that unity in the profession from which so much was to be expected.

### An Amendment.

Dr. Dunstan (Fulham) said he did not intervene in any spirit of opposition, but, in order to give greater effect to the resolution, he submitted an amendment deleting "on a trade union basis," and adding "and democratically organised as a trades union affiliated to the Labour Party and Trades Union Congress." Any faltering at this moment would be fatal to the organisation of the profession. The reason of the miners' success was that they were affiliated with the great Labour movement. He asked the meeting to take the next logical step and become part of the great trade union world. Mere registration as a trade union was not sufficient, and he spoke with a knowledge of such unions derived from the inside. He hoped the mover and seconder of the resolution would accept his amendment.

Capt. Morgan seconded the amendment, prefacing his remarks with "Mr. Chairman and fellow-slaves." He asked whether his hearers were prepared to ally themselves with the autocracy or with the democracy. He strongly urged them to follow thoroughly the democratic principle, and objected to the self-elected Medical Parliamentary Committee, which could not be called a democratic body.

Dr. Fielding-Ould said there was a very large body of opinion in the profession which was opposed to trade-union principles. The present resolution he regarded as revolutionary; it would split the profession as no other had ever done. There was a danger of being obsessed by the idea that the strike was the panacea for all evils. A golden image had been set up, and the profession was expected to bow down before it. Unity, not trade-unionism, was what the profession stood in need of, and he considered the only hope of securing what was asked for was in supporting the Medical Parliamentary Committee. (Dissent.) He had arrived at that conclusion after very careful consideration: he was not a member of it. After consultation with various members of the profession he felt sure that all sections would be well represented on that Committee.

(*Continued on page vi.*)

